# Dependence of the Startle Response on Temporal and Spectral Characteristics of Acoustic Modulatory Influences in Rats and Gerbils

**DOI:** 10.3389/fnbeh.2016.00133

**Published:** 2016-06-30

**Authors:** Natalie Steube, Manuela Nowotny, Peter K. D. Pilz, Bernhard H. Gaese

**Affiliations:** ^1^Institute of Cell Biology and Neuroscience, Goethe-University Frankfurt/MainFrankfurt, Germany; ^2^Institute of Neurobiology, University of TuebingenTuebingen, Germany

**Keywords:** acoustic startle reflex, prepulse inhibition, gap-prepulse inhibition, tinnitus, gap duration, background noise

## Abstract

The acoustic startle response (ASR) and its modulation by non-startling prepulses, presented shortly before the startle-eliciting stimulus, is a broadly applied test paradigm to determine changes in neural processing related to auditory or psychiatric disorders. Modulation by a gap in background noise as a prepulse is especially used for tinnitus assessment. However, the timing and frequency-related aspects of prepulses are not fully understood. The present study aims to investigate temporal and spectral characteristics of acoustic stimuli that modulate the ASR in rats and gerbils. For noise-burst prepulses, inhibition was frequency-independent in gerbils in the test range between 4 and 18 kHz. Prepulse inhibition (PPI) by noise-bursts in rats was constant in a comparable range (8–22 kHz), but lower outside this range. Purely temporal aspects of prepulse–startle-interactions were investigated for gap-prepulses focusing mainly on gap duration. While very short gaps had no (rats) or slightly facilitatory (gerbils) influence on the ASR, longer gaps always had a strong inhibitory effect. Inhibition increased with durations up to 75 ms and remained at a high level of inhibition for durations up to 1000 ms for both, rats and gerbils. Determining spectral influences on gap-prepulse inhibition (gap-PPI) revealed that gerbils were unaffected in the limited frequency range tested (4–18 kHz). The more detailed analysis in rats revealed a variety of frequency-dependent effects. Gaps in pure-tone background elicited constant and high inhibition (around 75%) over a broad frequency range (4–32 kHz). For gaps in noise-bands, on the other hand, a clear frequency-dependency was found: inhibition was around 50% at lower frequencies (6–14 kHz) and around 70% at high frequencies (16–20 kHz). This pattern of frequency-dependency in rats was specifically resulting from the inhibitory effect by the gaps, as revealed by detailed analysis of the underlying startle amplitudes. An interaction of temporal and spectral influences, finally, resulted in higher inhibition for 500 ms gaps than for 75 ms gaps at all frequencies tested. Improved prepulse paradigms based on these results are well suited to quantify the consequences of central processing disorders.

## Introduction

The acoustic startle response (ASR) of mammals is a fast contraction of facial and skeletal muscles evoked by a sudden and intense sound (Swerdlow et al., 1999). Stimulus and response are processed in the primary pathway of the ASR, a simple reflex-eliciting neuronal circuit containing only a few synapses located in the lower brainstem (Davis et al., 1982; Koch, 1999). Briefly, the cochlear root nucleus receives auditory nerve input and transfers startle-related activity to the caudal pontine reticular nucleus (PnC), which directly projects to relevant spinal motoneurons (Koch, 1999). Processing in the primary startle pathway is under the control of various modulatory influences from different areas of the brain, e.g., the pedunculopontine tegmental nucleus and laterodorsal tegmental nucleus reaching the primary pathway at the level of the PnC (Fendt et al., 2001). This is the neuronal basis for a variety of modulations of the magnitude of ASR behavior, most prominently the modulation by a non-startling prepulse presented shortly before the startle-eliciting noise. Depending on temporal arrangements of prepulse and startle stimulus, either enhancement of the startle response, called prepulse facilitation (PPF), or reduction of it, named prepulse inhibition (PPI), was found in rats (Reijmers and Peeters, 1994) and in mice (Plappert et al., 2004). Basically, short intervals (between prepulse and startle stimulus) up to 10 ms elicit PPF, whereas longer inter-stimulus intervals (ISIs) cause PPI in rats (Ison et al., 1973; Reijmers and Peeters, 1994). Comparable results were found in mice (Willott and Carlson, 1995).

These modulations of the ASR are widely used as a basis for behavioral tests to study neuronal mechanisms and neuropathological dysfunctions of sensorimotor information processing (Koch and Schnitzler, 1997). PPI is often disrupted in patients with neuropsychiatric disorders, like schizophrenia (Braff et al., 1978; Braff and Geyer, 1990), Huntington’s disease (Swerdlow et al., 1995), Tourette syndrome (Swerdlow and Sutherland, 2005) and auditory processing disorder (Moore, 2006). As the ASR can be elicited in rodents and humans in a comparable manner, rats and mice are seen as good animal models to study these disorders (Koch, 1999). Additional applications use inhibition of the ASR as a test paradigm in psychoacoustics. This has simplified studies in that field, as long periods of training are not necessary using this approach (Hoffman and Searle, 1965; Ison and Bowen, 2000) other than in classical approaches based on conditioned responses (e.g., Gaese and Wagner, 2002; Gaese et al., 2006). Lately, PPI was even used in complex acoustic discrimination experiments (Fitch et al., 2008). A more defined recent application is the objective tinnitus assessment by ASR measurements in rodents. To this end a method based on PPI of the ASR was developed using a gap of silence embedded in continuous background noise as prepulse (Gap-PPI) in rats (Turner et al., 2006). This gap-PPI method is based on the deficit of subjects with tinnitus, which cannot detect the gap when the simultaneously perceived tinnitus sound matches the background noise and thus fills in the gap, leading to less inhibition of the ASR. This gap detection technique is also applied in other rodents, like mice, gerbils and guinea pigs by now (Longenecker and Galazyuk, 2011; Nowotny et al., 2011; Dehmel et al., 2012).

In spite of the fact that the gap-PPI paradigm is widely used, several basic questions regarding its application are still open. As can be seen for tinnitus assessment, for example, the frequency-dependency of the gap-effect is still under investigation. Often gap-PPI levels were only high when measured with broadband noise (BBN) but not with narrowband noise (NBN) as background, which is necessary for tinnitus detection (Turner and Parrish, 2008; Ralli et al., 2010; Luo et al., 2014). Typically, studies tested 3–5 frequencies for tinnitus detection (Turner et al., 2006; Yang et al., 2007; Dehmel et al., 2012), but recently up to 10 frequencies were tested to characterize the tinnitus percept more precisely by scanning a substantial part of the hearing range (Nowotny et al., 2011). Otherwise it might be possible to miss the frequency of the tinnitus sound or further effects elicited by an acoustic trauma (Nowotny et al., 2011). In addition, frequency-dependent influences on ASR measurements that might corroborate the tinnitus determination using gap-PPI were recently described in mice (Longenecker and Galazyuk, 2012). Finally, the neural dysfunctions related to schizophrenia that were recently also linked to the auditory cortex (Kantrowitz et al., 2015), might very well be frequency-dependent. PPI-measurements used to characterize animal models of schizophrenia should therefore also take the spectral influence into account. Taken together, a closer look at frequency-dependency of the ASR, especially its modulation by PPI, should be beneficial.

Beside the spectral characteristics of the PPI modulation of ASR, influences of several temporal parameters are also not fully understood; specifically the dependence of startle inhibition on gap duration, which influences the degree of inhibition of a following startle response. PPI can be induced by prepulses of very different temporal characteristics. To date, mainly short-time interactions up to 100 ms were studied in PPI experiments (Ison and Bowen, 2000; Friedman et al., 2004; Ison et al., 2005; Swetter et al., 2010). Only recently, other applications of the PPI paradigm were developed using complex modulatory stimuli with comparably long (up to 1000 ms) duration. Lingner et al. (2013) used amplitude modulation as a prepulse during 1 s before the startle stimulus in gerbils and Floody et al. (2010) showed PPI by 100–300 ms long speech sounds as prepulses in rats. Significant differences in PPI-levels were shown even at these long stimulus durations. Temporal parameters in this long range might as well lead to optimized measurements using gap-PPI, as it is used for tinnitus assessment. This made us to investigate effects of long gap durations in PPI modulation, both, by itself, and in interaction with background frequency.

The present study examined in detail the dependence of the startle response on temporal and spectral characteristics of modulatory acoustic stimuli in rats and gerbils as these characteristics obviously influence methodologies based on ASR, which become more and more relevant especially for tinnitus assessment in rodents. Therefore, we performed PPI- and gap-PPI measurements with different prepulse and background noise frequencies in rats and gerbils. We found that PPI and gap-PPI are frequency-dependent in rats but not in gerbils. In addition, we compared inhibition values elicited by different gap durations and analyzed the effect of different noise-band widths on ASR. Long gap durations were found to produce stronger and more reliable inhibition of ASR.

## Materials and Methods

### Animals

In this study female Sprague Dawley rats, acquired from Charles River Laboratories (Sulzfeld, Germany), and Mongolian gerbils (*Meriones unguiculatus*), bred in-house, were used for comparison. Animals were kept on a 12/12 h dark/light cycle, with light phase starting at 7 am for the gerbils and at 7 pm for the rats. Food and water were available *ad libitum*. Experiments were carried out in accordance with the “Guide for the care and use of laboratory animals” (National Research Council, 1996) and with the Declaration of Helsinki and were approved by the local animal care committee. The different experiments were performed with six animal groups (3 gerbil, 3 rat) in total. Effects of gap duration on the ASR were studied in a group of 9 rats (2$\frac{1}{2}$ months old) with an average weight of 210.3 g (for results see Figure 4A). Effects of short gap durations (Figure 4B) and background noise (Figure 12) were investigated in a group of 16 gerbils (5 months old, 9 male/7 female) weighing on average 53.5 g. Effects of longer gap durations (Figure 4C) and background frequency (Figures 9–11) were examined in a group of 8 gerbils (2 male/6 female, 4 months old) and a group of 8 rats (2$\frac{1}{2}$ months old) with an average weight of 51.3 g and 207 g, respectively. Effects of frequency-dependency in noise-burst prepulse inhibition (NB-PPI; Figures 2, 3) and gap-PPI (Figures 5–8) were studied in a group of gerbils (*n* = 16, 9 male/7 female, 6 months old) and in a group of rats (*n* = 8, 4 months old) both weighing on average 55.9 g and 196.8 g, respectively. Due to technical problems, data from one rat were removed from one experiment (Figure 5, *n* = 7).

### Acoustic Startle Response Measurements

The ASR behavior was measured inside a sound attenuating booth (ENV-018SD, Med Associates St. Albans, VT, USA). This booth contained also a dimmed LED light (AmperLED AC806, Ampercell) and a video camera for monitoring during the experiments. The LED light was switched off for experiments with rats as they were tested during their active (dark) phase. Acoustic stimuli were generated using a custom-built computer program written in MATLAB (MATLAB R2007b, The MathWorks, Inc., MA, USA) that also recorded animal responses. The computer-generated stimuli were converted into analog signals using an external sound card (Fireface 400, RME, 24 bit, 96 kHz; Heimhausen, Germany). Waveforms were amplified (Rotel RB 1510, North Reading, MA, USA) and then delivered to a loudspeaker (HTH 8.7, Visaton, Haan, Germany), located 10 cm above the animal. The animal was sitting in a small, custom-built wire mesh cage (18 × 5.5 × 6.5 cm, for gerbils; 18 × 7 × 9 cm for rats) on a motion-sensitive platform (Med Associates Inc., St. Albans, VT, USA) containing a piezo element that converted animal movements into changes in electrical potential. The voltage output of the piezo element was amplified by a charging amplifier (PHM 250B; Med Associates Inc., St. Albans, VT, USA) and a measuring amplifier/filter-combination (custom-built, gain: 4), band-pass filtered at 1 Hz–3 kHz, and fed back into the sound card for recording. Four of these setups were installed in parallel in separate sound-attenuating booths, all attached to one sound card, so four animals could be tested simultaneously. Calibration of the frequency response of the sound system was performed using a 0.25″ condenser microphone (Type MK301, Microtech Gefell) and a measuring amplifier (Model 2609, Bruel and Kjaer). The sound pressure level for each stimulus component used in the study (startle stimulus, noise-burst prepulses, background noise-bands, background pure-tones) was calibrated separately (i.e., not simply derived from the frequency response). To guarantee low animal spontaneous movements, all animals were acclimated to the setup before starting the experiments. This was done by positioning the animal in the cage for 10 min on three consecutive days, while neither stimulus nor background noise was presented.

The dependency of the ASR on the sound pressure level (SPL) of the stimulus was determined as an input/output (I/O) function of ASR. Before stimulus presentation, animals were adapted for about 10 min to the test cage. To reduce short-term habituation during measurements, eight startle stimuli of 105 dB SPL (peak-to-peak) were then presented, which were not used for evaluation (Gaese et al., 2009). Then, startle stimuli with varying SPL (65–115 dB SPL, 10 dB steps) were presented in pseudorandom order with an inter-trial interval (ITI) of 7 s. The startle stimulus, which was the same for all experiments, was a 20 ms long (BBN: 1.5–20 kHz) with a rise/fall time (r/f) of 0.0001 ms. Each stimulus was repeated 20–25 times, resulting in 120–150 evaluated stimulus presentations. The overall duration of one session was about 20 min.

### Prepulse Inhibition

**Figure 1:**
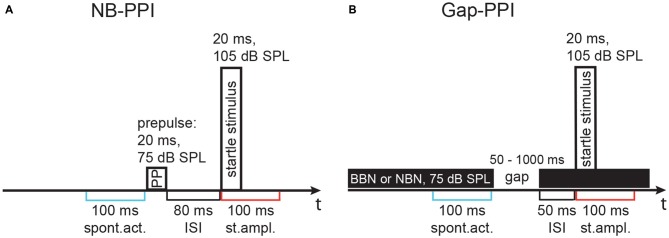
**Schematics of the stimulus configuration in two paradigms using different types of prepulses.** Stimulus configurations are shown above the time line, time windows for activity measurement are indicated below. **(A)** Noise-burst prepulse inhibition (NB-PPI) was tested with a noise prepulse (narrowband noise, NBN, of 0.5 octaves, oct, width around variable center frequencies, 75 dB SPL, 20 ms duration) that was followed by the startle-eliciting stimulus (broadband noise, BBN, burst: 1.5–20 kHz, 105 dB sound pressure level, SPL, 20 ms duration) after an inter-stimulus interval (ISI) of 80 ms. **(B)** Gap-prepulse inhibition (gap-PPI) was tested in trials with background noise (either broadband or a 0.5-oct wide band of noise) with a gap included as a prepulse (duration 50–1000 ms) that was followed by the same startle-eliciting stimulus after an ISI of 50 ms in most cases. An ISI of 0 ms, however, was also used with very short gaps as indicated in Figure 4. Spontaneous activity (spont. act.) and the startle response amplitude (st. ampl.) were measured in 100 ms time windows as indicated in blue and red, respectively, below the timelines in **(A,B)**.

The modulatory influence of a prepulse on the startle response was determined, first by using a noise-burst prepulse to determine inhibitory effects (NB-PPI), and second by using a gap-in-noise prepulse to measure gap-PPI. After adapting the animals for 10 min to the test cages, eight startle stimuli were presented for habituation and stabilization of the startle response values, which were discarded from evaluation. Then, NB-PPI was measured by comparing the ASR induced by startle stimuli with a preceding noise-burst prepulse against responses to the startle stimuli alone. The prepulses had a duration of 20 ms and a sound pressure level of 75 dB SPL. The r/f was 1 ms and the ISI from offset of prepulse to onset of startle stimulus was always 80 ms. Noise-burst prepulses were either NBN (bandwidth: 0.5 octaves, oct) with varying center frequency between 4 and maximal 30 kHz or BBN (1.5–20 kHz). The detailed stimulus configuration is shown in Figure 1A. Noise-burst prepulses were presented in pseudorandom order. The ITI was always 7 s long. Each prepulse condition was repeated 16 times, while the control startle stimulus without prepulse appeared twice as often. One session lasted in total about 30 min.

For gap-PPI measurements, animals were again adapted to the test cages for 10 min and eight startle stimuli were presented, which were not used for evaluation. Afterwards, startle stimuli alone or preceded by a short gap, both embedded in background noise of 75 dB SPL, were presented. The ITI was always 14 s. The background noise was in total 10 s long, being either NBN centered at different frequencies between 4 and 20 kHz with a bandwidth of 0.5 oct or BBN (Figure 1B). The bandwidth was 0.25 oct in only one gap-PPI experiment with gerbils (see Figure 12). In an additional gap-PPI experiment with rats, pure-tones between 4 and 32 kHz were used as acoustic background. Several experiments with varying gap durations were performed. First, gap durations embedded in BBN were varied between 2 and 1000 ms for gerbils and 2 and 500 ms for rats with 1 ms r/f (ISI = 0 ms) or 0.1 ms r/f (ISI: 25–50 ms). Then, in gerbils and rats gap durations of 50–1000 and 75–500 ms, respectively, were measured with different background noise bands. To determine gap-PPI, trials with and without gap were presented for each of the different background noises. Every trial type was repeated 16 times in pseudorandom order. The resulting 160 trials in total led to a duration of about 40 min for each session. Measurements were repeated when the inhibition was less than 30% or higher than 90% indicating a high level of spontaneous motor activity in individual animals directly before the startle stimulus. NB-/gap-PPI measurements of rats were split into two separate sessions, when the number of frequencies tested exceeded five, so that the duration of one session never lasted longer than 45 min. In these cases, the second session was measured 3 h in minimum and 24 h in maximum after the first session to avoid habituation effects (random order of frequencies).

### Data Analysis

The absolute startle response was determined as the maximum peak-to-peak response amplitude measured in a 100-ms time window after the onset of the startle stimulus (Figure 1). Preceding the onset of the startle stimulus, spontaneous motor activity was determined as the maximum peak-to-peak amplitude in a time window of 100 ms before the onset of the prepulse or the gap-in-noise. Response strength for I/O and PPI was calculated by subtracting the maximal amplitude of the spontaneous motor activity from the maximal amplitude of the startle response. If the spontaneous activity of an animal was too high and the resulting value was lower than −0.4 V it was not used for evaluation (occurred in less than 3% of the trials). Subsequently, the mean of all values per stimulus type and animal was calculated.

The amount of response modulation or PPI was quantified by subtracting the startle response with prepulse from the startle response without prepulse divided by the startle response without prepulse multiplied by 100:

Inhibition (%) = (startle response without prepulse−startle response with prepulse)/startle response without prepulse * 100.

Average values from all test animals were plotted as mean ± standard error (SEM) as a function of prepulse frequency or background frequency (NB-PPI, gap-PPI).

Statistical analysis was done using the statistical software JMP (Version 7.0; SAS Institute Inc., Cary, NC, USA). For each animal, averages of inhibition, startle amplitude or spontaneous motor activity were analyzed in one- and/or two-way ANOVAs with separate factors of test frequency, gap length or bandwidth. *Post-hoc* analysis was done using Tuckey HSD or independent contrasts corrected for multiple comparisons with the Bonferroni-Holm procedure. Significance levels are indicated in figures as *p < 0.05, **p < 0.01 and ***p < 0.001.

## Results

We investigated the influence of temporal and spectral characteristics of modulatory acoustic stimuli in rats and gerbils on the ASR. While in the first part data on the startle response after noise-burst prepulses are presented, the second part presents data on the modulatory effects by gap-prepulses. This second part includes the importance of temporal parameters of gap-prepulses, then results on frequency-dependency, and finally, the importance of the interaction between temporal and spectral characteristics. Data from three different groups of rats and three gerbil groups were included in this study (see “Materials and Methods” Section) in order to provide a broad picture of the influences studied.

### Spectral Influences on NB-PPI: Frequency-Dependency in Rats But not in Gerbils

The frequency contents of noise-burst prepulses used in PPI measurements differ between labs and questions asked. Here, we systematically investigated the influence of the center frequency of noise-burst prepulses on the ASR over a broad range of test frequencies.

**Figure 2:**
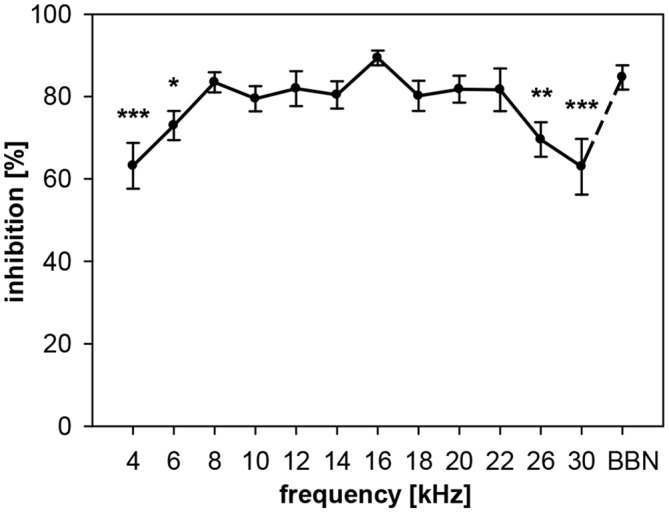
**Noise-burst PPI in rats: dependency on prepulse frequency.** Noise-burst prepulse inhibition (NB-PPI) was tested for noise-burst prepulses (onset 100 ms before the startle stimulus, 20 ms duration, 75 dB SPL) of either narrow-band noise (0.5-oct wide) around 13 different center frequencies between 4 and 30 kHz (as indicated on the *x*-axis) or BBN (1.5–20 kHz). Inhibition (NB-PPI) is given as the percentage of response reduction relative to the response without prepulse. Average inhibition (*n* = 8, Mean ± SEM) depended on the frequency tested (ANOVA). Significant differences compared to inhibition at BBN occurred at low and high center frequencies (*Post-hoc* test: *p < 0.05, **p < 0.01, ***p < 0.001).

In rats, NB-PPI depended significantly on the center frequency of narrowband (0.5 oct) prepulses (ANOVA: *R*^2^ = 35%, *F*_(12,91)_ = 4.1, *p* < 0.0001). While the amount of inhibition was almost constantly high (80–90%) for frequencies in the range from 8 to 22 kHz, inhibition was lower below this frequency range (8 kHz) and above (22 kHz; Figure 2). The lowest inhibition values of about 63% were measured at center frequencies of 4 and 30 kHz. The highest inhibition value of 89% was found at 16 kHz. This value at 16 kHz was not significantly different to PPI induced by BBN-prepulses (84%), the commonly used type of prepulse in the literature.

**Figure 3:**
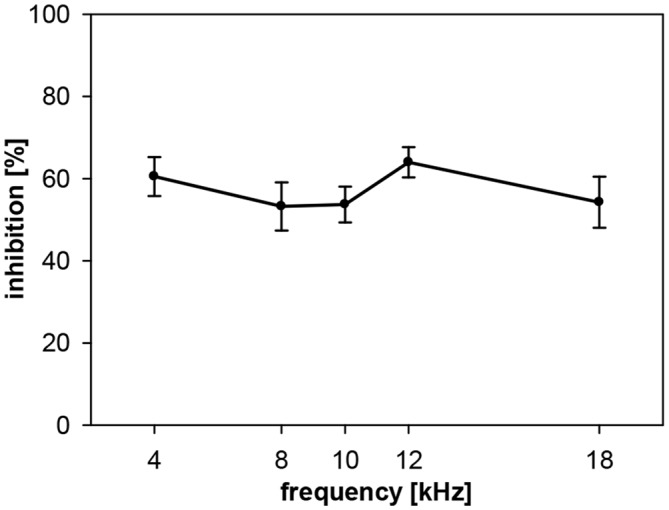
**Noise-burst PPI in gerbils: dependency on prepulse frequency.** NBN prepulses (onset 100 ms before startle stimulus, 20 ms duration, 75 dB SPL, 0.5-oct wide) with five different center frequencies (4, 8, 10, 12 and 18 kHz) were presented shortly before the startle stimulus (105 dB SPL). Inhibition (NB-PPI) is given as the percentage of response reduction relative to the response without prepulse. Average inhibition (*n* = 16, mean ± SEM) did not depend on the frequencies tested.

The PPI by noise-bursts in gerbils, on the other hand, was not depending on prepulse frequency in the frequency range tested, which was smaller (4–18 kHz) than the range tested in rats, though. Gerbils showed a quite constant inhibition of about 60% (Figure 3). The main difference to the curve measured in rats is the undiminished PPI at 4 kHz. However, average NB-PPI in gerbils (57%) was lower than PPI in rats (78%; *F*_(1,110)_ = 63.5, *p* < 0.0001).

### Temporal Aspects of Gap Duration: Facilitation Occurs Not in All Rodents

Gaps in background noise have a strong modulatory influence on the strength of a following startle response. Gap-prepulses can have strongly varying duration in different functional contexts such as tinnitus determination or psychophysical threshold estimation. This difference impairs comparisons on temporal aspects of gap-PPI. Therefore, we systematically measured gap-PPI in rats and gerbils for gap durations from 2 to 1000 ms.

**Figure 4:**
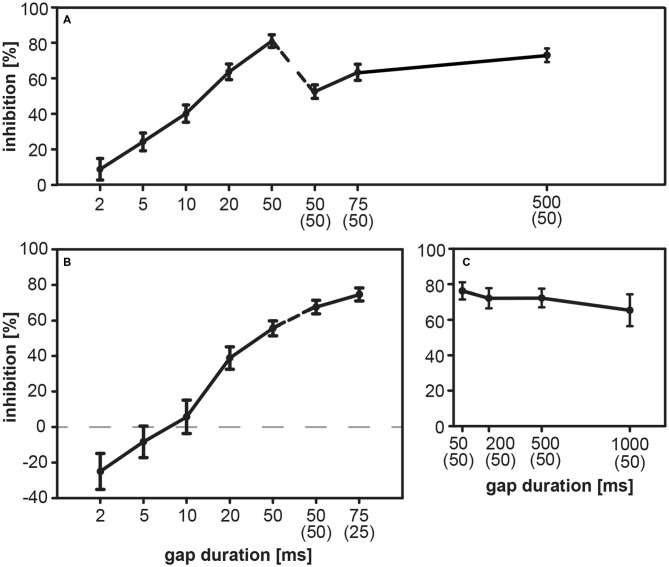
**Gap-PPI in rats and gerbils: influence of gap duration.** The acoustic startle response (ASR) was modulated by gaps in BBN background in rats (*n* = 9; part **A**) and in gerbils (group 1, *n* = 16, part **B**; group 2, *n* = 8, part **C**). Gap-PPI is shown as a function of gap duration of gaps either preceding the startle stimulus directly or with a substantial ISI as indicated in parentheses below gap duration. **(A)** Rats showed increasing mean inhibition with longer gap durations. Introducing a 50-ms interval between gap offset and startle stimulus onset slightly reduced the amount of inhibition. **(B)** Gerbils also showed an overall increase of inhibition of the startle response with longer gap durations. Only in gerbils the startle response was even facilitated (negative values of gap-PPI) by short gaps directly preceding the startle stimulus with gap durations up to 5 ms. Longer gaps (above 10 ms) inhibit the ASR. **(C)** Mean inhibition (gap-PPI) in gerbils by longer gaps with an ISI of 50 ms was high even up to durations of 1000 ms. Data are given as the percentage of response reduction relative to the response without gap-prepulse. Average inhibition values are shown as mean (± SEM).

Modulatory influences of gaps embedded in BBN background were determined in the rat for short gap durations (2–50 ms) ending directly before the startle stimulus and for long gap durations (50–500 ms) with an ISI of 50 ms. The shortest gap duration of 2 ms showed hardly any effect on the magnitude of ASR, but from 5 ms on the silent gap reduced the magnitude of ASR causing gap-PPI (Figure 4A). Inhibition increased with increasing gap duration from 24% at 5 ms up to 81% at 50 ms. This was also true for gaps ending 50 ms before the startle stimulus, although these gaps induced lower inhibition than 50 ms-gaps ending directly before the startle stimulus.

In contrast, gerbils showed gap-PPF for short gaps up to 5 ms before the acoustic startle stimulus (Figure 4B). Gap durations above 10 ms are accompanied with increasing inhibition for longer gap durations up to 75 ms (Figure 4B). An application of an ISI between gap and startle stimulus increased gap-PPI in gerbils. The maximum gap-PPI value was 75%. Gap-PPI remained constantly at high levels between 65 and 76% for longer gap durations up to 1000 ms in gerbils (Figure 4C).

### Spectral Influences on Gap-PPI in Rats: Frequency-Dependency not with Pure-Tones

**Figure 5:**
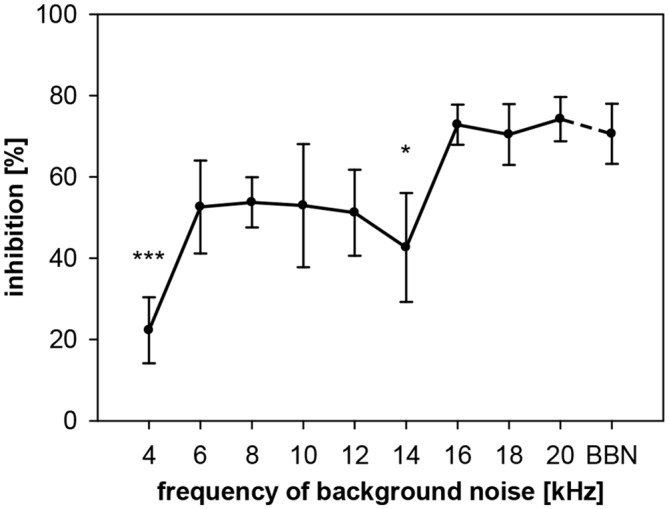
**Gap-PPI in rats: dependency on center frequency of the background noise.** Inhibition was induced by 500 ms long gaps embedded in background noise (75 dB SPL) that was 0.5 oct wide and centered at nine different frequencies from 4 to 20 kHz. BBN was tested in addition. Center frequency influenced mean inhibition (*n* = 7, ±SEM), with gap-PPI at 4 kHz and 14 kHz significantly below gap-PPI at BBN. (*Post-hoc*: independent contrasts; *p < 0.05, ***p < 0.001).

To describe the dependency of gap-PPI on the frequency of the acoustic background in rats, we conducted two experiments. In the first one, gaps in NBN with different center frequencies were studied. In the second experiment, we used pure-tones to study frequency influences. Using noise, we measured gap-PPI with background noise bands around different center frequencies (4–20 kHz in 2 kHz-steps; spectral width: 0.5 oct) or BBN, with 500-ms gaps as prepulses. Gap-PPI in rats depended on the frequency of the background noise (*F*_(9,120)_ = 2.6, *p* = 0.0087, Figure 5). Inhibition was on average around 70% in the high frequency range (16–20 kHz). At lower frequencies between 6 and 14 kHz the inhibition was around 50%. The lowest value for gap-PPI with less than 20% inhibition was measured at 4 kHz. Gap-PPI at 4 and 14 kHz was significantly different from BBN. Gap-PPI for broadband background noise, was at a high level (71%), as known from the literature.

**Figure 6:**
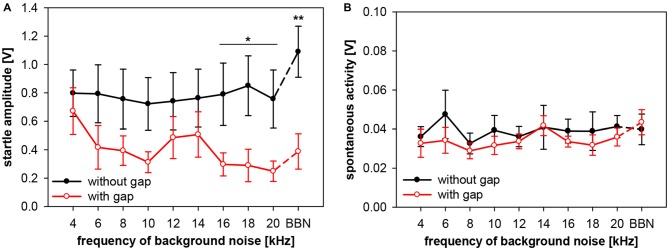
**Startle amplitude and spontaneous motor activity during gap-PPI stimulation in rats: dependency on center frequency of the background noise.** Average absolute startle amplitude **(A)** and spontaneous motor acitvity **(B)** of trials with 500-ms gap (red open circles) and without gap (black closed circles) are shown for background NBNs (0.5 oct) of different center frequencies and for BBN. For the startle amplitude **(A)**, the background noise frequencies with significant differences between gap and no-gap trials are indicated (*Post-hoc*: independent contrasts; *p < 0.05, **p < 0.01). Mean values (*n* = 7) are given with ±SEM.The *y*-axes differ in scaling between **(A,B)** because of much lower spontaneous motor activity values compared to startle amplitude.

Frequency-dependency of gap-PPI, as shown above, can have various possible sources. Either the no-gap trials (startle-only-in-noise), or the trials including gap-prepulse, or both could in general be the source of spectral influences. In addition, hugh variations in spontaneous motor activity could cause frequency-dependent effects as well. Therefore, we analyzed (in separate 2-way ANOVAs) startle amplitude and spontaneous motor activity of gap-PPI measurements in both types of trials, with and without gap, for the influences of frequency and gap vs. no-gap. Analysis of startle amplitudes revealed no main effect of frequency (*F*_(9,120)_ < 1, n.s.), but a significant difference in the gap- vs. no-gap comparison (*F*_(1,120)_ = 34.7, *p* < 0.0001; Figure 6A). The startle amplitude in gap trials was significantly reduced compared to no-gap trials in the high frequency range between 16 and 20 kHz as well as at BBN (*Post-hoc*: see indication in Figure 6A).

Further detailed analysis revealed that startle amplitudes of no-gap trials (Figure 6A, black symbols) were not depending on the center frequency of the background noise (*post-hoc*: independent contrasts) and therefore a constant basis for gap-PPI. This indicates that only different amounts of inhibition at different background frequencies caused the frequency-dependency of gap-PPI (Figure 5). The comparably large error bars (SE) indicate strong individual differences between animals. Spontanous motor activity, measured in a time window right before the gap was very low compared to the startle response measured (Figure 6B; note different scaling of the amplitude compared to Figure 6A) and can also be excluded as source of frequency-dependency since both values (with and witout gap) are very low and not different from each other (*F*_(1,120)_ = 2.3, *p* = n.s.).

**Figure 7:**
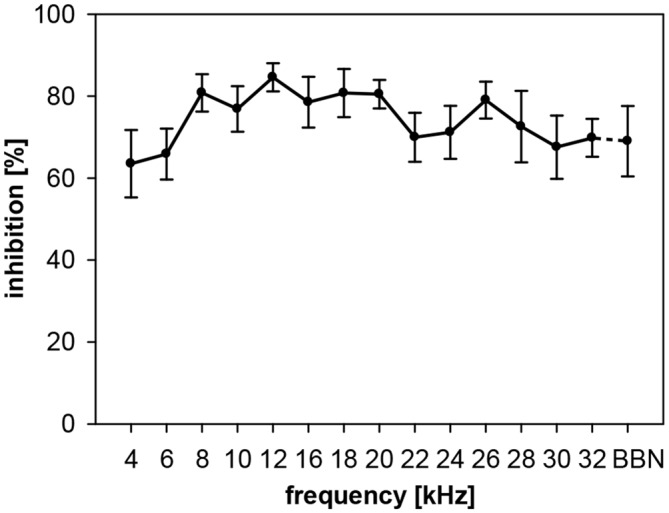
**Gap-PPI in rats: dependency on the frequency of background pure-tones.** Inhibition was induced by 500-ms long gaps embedded in pure-tone background (75 dB SPL). Gap-PPI was measured at 14 different frequencies between 4 and 32 kHz (omitting 14 kHz). For comparison, gaps in BBN were also tested. Average inhibition (*n* = 8, ±SEM) did not depend on the background frequencies tested (see main text).

A tonal background for gap-PPI measurements was tested in addition, to compare results with the mainly used gap-in-noise stimuli. We tested the influence of gaps in pure-tone backgrounds at different frequencies from 4 to 32 kHz (in 2 kHz steps, omitting 14 kHz, gap duration 500 ms) compared to a BBN background. There was no main effect of frequency (1-way ANOVA: *F*_(14,105)_ = 1.1, n.s.) over a rather broad frequency range. The inhibitory effect of average gap-PPI in pure-tone background in rats was constantly high in the range between 65 and 85% for all frequencies tested (Figure 7). By comparing data from tonal vs. noise-band background (Figure 5 vs. Figure 7) we found that gap-PPI was on average higher for pure-tone background (75%) than for 0.5-oct broad background noise (57%, Figure 5), when using 500-ms gaps in both conditions (2-way ANOVA: *F*_(1,108)_ = 23, *p* < 0.0001; only frequencies tested under both conditions were included). On the other hand, gap-PPI in pure-tone background was not significantly different to gap-PPI in broadband background noise.

### Spectral Influences on Gap-PPI in Gerbils: No Frequency-Dependency Observed

**Figure 8:**
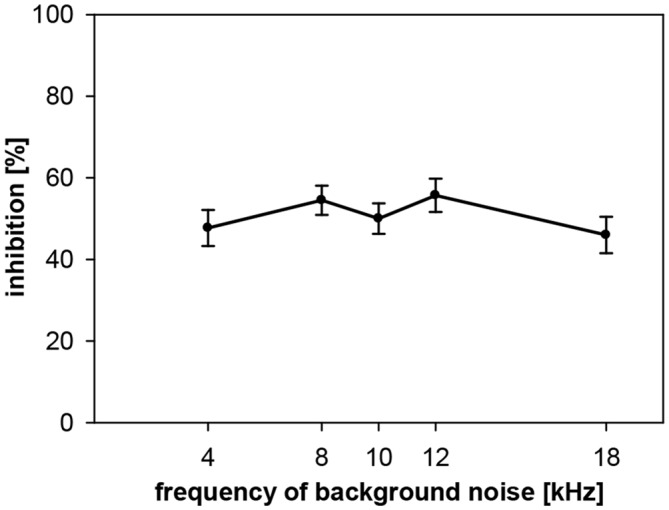
**Gap-PPI in gerbils: dependency on center frequency of background noise.** Inhibition was induced by 500-ms long gaps in narrowband background noise (75 dB SPL, 0.5 oct wide) around five different frequencies (4, 8, 10, 12 and 18 kHz). Average inhibition (*n* = 16, ±SEM) did not depend on the frequencies tested.

Gap-PPI was also measured in gerbils with background noise bands around five different center frequencies (4, 8, 10, 12 and 18 kHz; bandwidth 0.5 oct) for a gap duration of 500 ms. Average gap-PPI was about 50% in the measured frequency range in gerbils (Figure 8). This was as high as gap-PPI in the middle frequency range (8–12 kHz) measured in rats but lower than gap-PPI in the higher frequency range (16–20 kHz) in rats. Thus, different from rats, gap-PPI was not depending on background noise center frequency in gerbils.

### Spectro-Temporal Interactions on Gap-PPI in Rats: Stronger Gap-PPI With Longer Gaps

**Figure 9:**
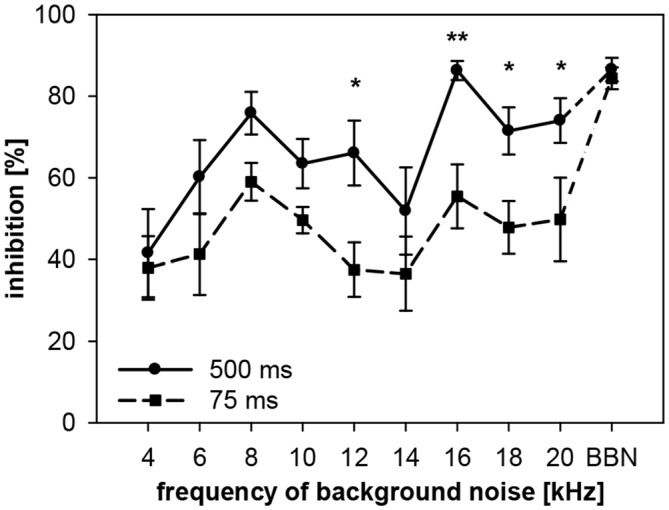
**Gap-PPI in rats: dependency on the interaction between center frequency of background noise and gap duration.** Inhibition was induced by 500-s long (circles) and 75 ms short gaps (squares) in narrowband background noise (75 dB SPL, 0.5 oct) at nine different center frequencies (4–20 kHz) as well as in BBN. Average gap-PPI (*n* = 8, ±SEM) for both, long and short gaps, depended on noise frequencies tested. Significant differences between curves (indicated by asterisks above the curves) occurred mainly at high frequencies. (*Post-hoc*: independent contrasts; *p < 0.05, **p < 0.01).

To examine the interaction between spectral and temporal parameters, the dependency of gap-PPI on gap duration and on spectral width of the background noise was tested simultaneously in rats. Gap-PPI was measured using short (75 ms) and long (500 ms) gaps-in-noise bands (0.5 oct) around different center frequencies (4–20 kHz in 2 kHz-steps) or BBN. Gap-PPI in rats was dependent on gap duration (main effect in a 2-way ANOVA testing influences of gap duration and frequency; *F*_(1,140)_ = 30.1, *p* < 0.0001; Figure 9) as well as on noise frequency (*F*_(9,140)_ = 6.9, *p* < 0.0001). Inhibition was on average higher for 500-ms gap duration (68%) than for 75 ms (50%). This difference in gap-PPI between gap durations was more pronounced in the higher frequency range between 12 and 20 kHz: the four significant frequency-specific differences in inhibition between gap-durations were among the five highest frequencies tested, as indicated in Figure 9. The interaction of frequency by gap duration in the 2-way ANOVA, however, was not significant (*F*_(9,140)_ < 1, n.s.). In spite of that, curve progression for gap-PPI was quite parallel between long and short gap durations over the frequency range tested. For both, 500- and 75-ms gap duration, the lowest inhibition was measured at 4 kHz (42% and 38%, respectively) and again at 14 kHz (52%, 37%). Mentionable is the difference in the degree of variance of the standard error between the curves for 75- and 500-ms gap duration. At a given frequency, the variance of gap-PPI was mostly larger for 75-ms gap duration. For BBN, however, the inhibition was less variable and again reliably high for both gap durations (84% and 86%, respectively).

**Figure 10:**
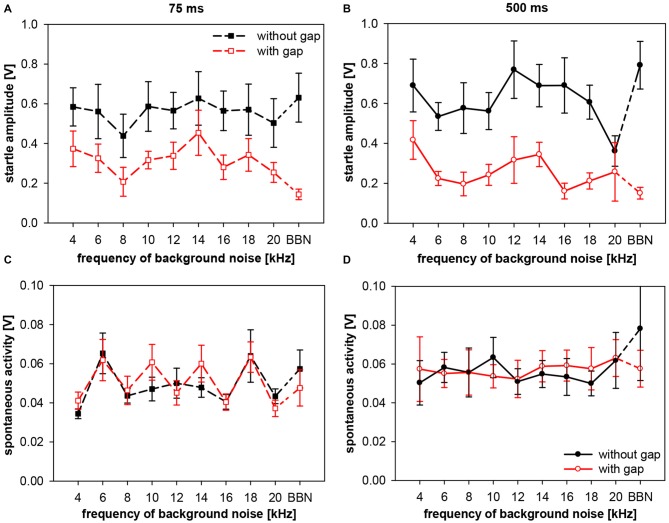
**Startle amplitude and spontaneous motor activity during gap-PPI stimulation in rats: dependency on the interaction between center frequency of background noise and gap duration.** Absolute startle amplitude **(A,B)** and spontaneous motor acitvity **(C,D)** of gap trials (red symbols) and no-gap trials (black symbols) are shown for narrowband background noise (75 dB SPL, width 0.5 oct) of different center frequencies (4–20 kHz) as well as in BBN. Average values (*n* = 8, ±SEM) from gap-PPI measurements with 75-ms long gaps (left panels) and with 500-ms long gaps (right panels). Note that the scaling of the *y*-axes differs between upper **(A,B)** and lower **(C,D)** panels.

These data on the interactive influences of gap duration and frequency on the amount of gap-PPI were investigated in greater detail by also analyzing the underlying absolute startle response amplitudes and the spontaneous motor activities. The aim was to determine possible components in the startle behavior leading to the observed differences in gap-PPI. Therefore, we analyzed (in separate 3-way ANOVAs testing influences of frequency, gap duration and gap vs. no-gap) startle amplitude and spontaneous motor activity of gap-PPI measurements, with two different gap durations (75 and 500 ms). Analysis of startle amplitudes revealed only a significant main effect for comparisons between trials with and without gap (*F*_(1,280)_ = 107.1, *p* < 0.0001). The other main effects (frequency and gap duration) as well as their interactions were not significant. This implies that startle amplitudes in no-gap trials (Figures 10A,B; black symbols) were, as expected, not significantly different between short (75 ms) and long (500 ms) gap durations (*post-hoc*: Tukey HSD; n.s.).

In gap-trials, however, corresponding absolute startle amplitudes (i.e., same frequency) after 500-ms gaps were mostly lower than after 75-ms gaps, mirroring the above shown difference in gap-PPI. As a consequence, startle amplitude for 500-ms gaps was significantly different between gap and no-gap trials at all frequencies tested except 20 kHz (*Post-hoc*: independent contrasts), whereas for 75-ms gaps this was only the case for 16 kHz and BBN (*Post-hoc*: independent contrasts). This clearly indicates towards a difference in the amount of inhibition between short and long gap durations (Figure 10). Interestingly, the graphs showing startle amplitudes with and without gap are quite parallel in each of the panels for short (Figure 10A) and long (Figure 10B) gap durations. Spontaneous motor activity was not significantly different between short (75 ms) and long (500 ms) gap durations (3-way ANOVA: *F*_(39,280)_ < 1, n.s.) and not significantly different between trials with and without gap for both gap durations (both *F*_(1,280)_ < 1, n.s.). This indicates that the measured differences in gap-PPI between gap durations (Figures 10C,D) were not caused or affected by differences in spontaneous motor activity.

### Spectro-Temporal Interactions on Gap-PPI in Gerbils: Only Width of Background Noise Influences Gap-PPI

**Figure 11:**
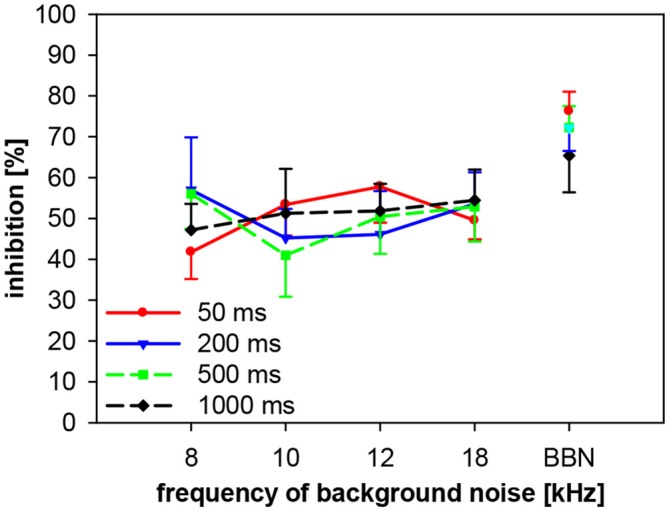
**Gap-PPI in gerbils: dependency on the interaction between center frequency of background noise and gap duration.** Gap-PPI was induced by 50, 200, 500 and 1000 ms-long gaps embedded in narrowband background noise (75 dB SPL, 0.5 oct wide) at four different center frequencies (8, 10, 12 and 18 kHz) as well as BBN. Mean gap-PPI (*n* = 8, ±SEM) by different gaps was in the same range but gap-PPI with BBN was always higher than with NBN.

We also investigated the importance of spectro-temporal interactions of gap-PPI in gerbils, using four different gap durations between 50 ms and 1000 ms. Gaps were embedded in background noise bands of four different center frequencies (8, 10, 12 and 18 kHz) and in BBN. Gap-PPI depended neither on gap duration nor on frequency of background noise and their interaction was also not significant (2-way ANOVA; both, gap duration and frequency: *F*_(3,96)_ < 1, n.s.; interaction: *F*_(9,96)_ < 1, n.s.; Figure 11). At all frequencies tested the inhibition was in the same range (40–60%) for gap durations between 50 and 1000 ms in gerbils, but it was on average always higher with BBN (68%) compared to NBN (48%).

### Minor Influences and Comparability of Startle Measurements in Gerbils

**Figure 12:**
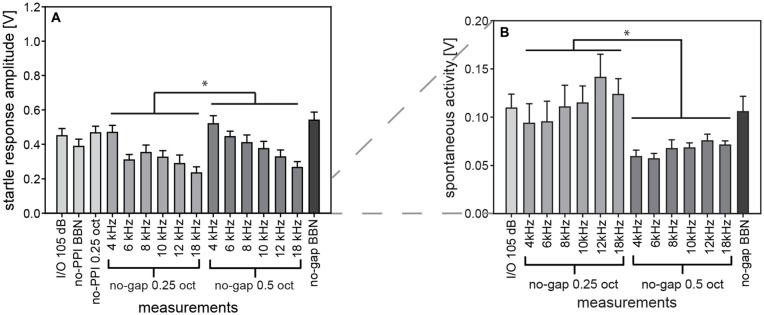
**Comparing startle response amplitudes in different test paradigms in gerbils.** Comparable measurements of absolute startle amplitude **(A)** and spontaneous motor acitvity **(B)** taken from repeated test sessions over a time period of 2 months in the same group of gerbils are shown to investigate additional influences on the ASR behavior. Specifically, data from trials without prepulse are shown for Input-Output (I/O) function, PPI- and gap-PPI. While in all of these measurements the same startling stimulus of 105 dB SPL was presented, the acoustic background was either silent (for I/O measurements and no-PPI), or NBN of 0.25 oct or of 0.5 oct width (as indicated by the labels in the *x*-axes), or BBN. Average values (*n* = 16, ±SEM) for gerbils are shown. Startle amplitudes with 0.5-oct NBN were higher than with 0.25-oct NBN and spontaneous activity was lower with 0.5-oct NBN than with 0.25-oct NBN (*p < 0.05). Note that the *y*-axes differ between panels.

Investigations on the ASR in mammals have a long tradition and were mainly performed in rodents, specifically rats and mice. Startle response in gerbils, on the other hand, has only recently been investigated and is now being used in tinnitus research (Nowotny et al., 2011; Kiefer et al., 2015). Compared to rats and mice, only few data are available in gerbils regarding confounding influences on startle measurements, especially when animals are involved in repeated experimental sessions where they are exposed to hundreds of startle stimuli. A confounding problem could be long-term habituation as it has been described for rats (Davis and Wagner, 1968; Leaton, 1976). Therefore, we analyzed for gerbils the startle amplitude and spontaneous motor activity from several experimental sessions that included similar types of stimuli (Figure 12). Firstly, we compared trials with the same startle stimulus from I/O-function experiments and PPI measurements. These measurements were compared to determine the influence of long-term habituation. Secondly, gap-PPI-measurements also include trials without prepulse in background noise. Since they had the same startling stimulus (105 dB SPL) these measurements were used to characterize the influence of spectral width and of center frequency.

We assume that long-term habituation had no significant influence on the repeated measurements over a period of 2 months, since startle amplitudes of the first measurement (I/O- function, Figure 12A) were not significantly different from that of the last measurement and from comparable measurements in between (1-way ANOVA comparing “I/O 105 dB” “no-PPI BBN” “no-PPI 0.25 oct” and “no-gap BBN”: *F*_(3,60)_ = 2.2, n.s.). Thus, repeated measurements over a long time period are possible, as startle amplitude was consistently stable in gerbils.

The background noise of our measurements was varied in spectral width (NBN: 0.5 or 0.25 oct) and in center frequency (4, 6, 8, 10, 12, 14 and 18 kHz) in gerbils. Their influences on startle amplitude were found to be significant (2-way ANOVA; main effect of spectral width: *F*_(1,180)_ = 6.7, *p* = 0.0102; main effect of frequency: *F*_(5,180)_ = 8.3, *p* < 0.0001). Startle amplitudes were higher with 0.5-oct than with 0.25-oct NBN (Figure 12A) and they significantly decreased with center frequency of the background noise band (*post-hoc*: Tukey HSD).

The spontaneous motor activity was also influenced by background noise. Activity levels depended on spectral width of NBN (2-way ANOVA; main effect of NBN width: *F*_(1,180)_ = 44.3, *p* < 0.0001) and were significantly lower during 0.5-oct noise compared to during 0.25-oct noise (Figure 12B). Frequency, on the other hand, had no significant influence (*F*_(5,180)_ = 1.9, n.s.). Therefore, the acoustic background noise might have influenced the state of excitation of gerbils, thereby modulating the basis for different measurements. However, a variation depending on individual daily condition is also possible, since spontaneous motor activity was only lower with 0.5-oct background noise.

## Discussion

The aim of the present study was to better define the stimulus situation inducing PPI in rats and gerbils. Therefore, several temporal and spectral stimulation parameters of noise background and prepulse stimuli influencing the ASR were analyzed, such as gap duration, spectral width of background noise and background frequency. This leads to a better understanding of the behavioral components underlying PPI and could allow for a more directed and optimized application of PPI paradigms in areas such as tinnitus research.

### Gap-PPI Increases With Gap Duration in Rats and Gerbils

In both species investigated, gap-PPI in BBN mainly increased with an increase in gap duration. This increase of gap-PPI with increasing gap duration is in line with similar studies in rats (Zou et al., 2007; Sun et al., 2011) and in mice (Walton et al., 1997; Weible et al., 2014), although gaps in these studies ended 50–60 ms before the startle stimulus. In gerbils, gaps had to be longer than 10 ms to induce strong inhibition, which is comparable to findings from PPI measurements in rats (Reijmers and Peeters, 1994) and mice (Plappert et al., 2004).

A species-specific difference, however, was found for very short durations. In rats, gap prepulses of 2 ms duration had hardly any effect on startle amplitude, leading to the assumption that such a short gap was little noticed by rats. Gerbils, on the other hand, had a different behavioral response and showed facilitation for short gaps of 2–5 ms duration. This is comparable to young rats, in which very short gaps also had a facilitatory effect. This facilitation in rats, however, was not very stable and diminished over repeated testing on three consecutive days (Swetter et al., 2010).

Analyzing long gaps, we found that for durations longer than about 50 ms, gap-induced inhibition was at about the same level up to at least 500 ms in rats and gerbils. Therefore, long gap durations can be used in gap-PPI measurements, and obviously induce stable inhibition. Such strong influences on the startle response occurring as early as 500 ms before the startling response are consistent in timing with startle-modulating changes in more complex stimulus arrangements described in the literature (e.g., Floody et al., 2010; Lingner et al., 2013). While all effects on the ASR in this long time window are from the auditory domain, the neuronal basis is unclear. The modulatory input to the startle pathway originating from the pedunculopontine tegmental nucleus was described as a cholinergic inhibitory influence with rather long time constant (Bosch and Schmid, 2008). A more central modulation of sensorimotor processing such as building up a working memory trace in the auditory cortex can serve as an alternative explanation (Sakurai, 1994). Still, some kind of sensory memory might be a likely candidate, although reverberating activity of this temporal extend has not been described so far in the rodent auditory pathway.

Such very long gaps, however, open the possibility for rather different interpretations of the dependency on gap duration. Other than just having a gap in noise before the startle stimulus one might see the stimulus arrangement as some early, long noise (before the gap) ending abruptly, followed after a delay (the gap) by a noise-burst prepulse right before the startle stimulus that might lead to different degrees of NB-PPI. This second pulse should be more prominent the longer the duration of the gap is, presumably for durations longer than 50 ms. While our rat data support this idea with a slight increase in PPI to some degree (see Figure 4A), the gerbil data show a reduction in PPI with gap duration above 50 ms (Figure 4C). Further experiments comparing gap-PPI with a substantial ISI (e.g., 50 ms of noise right before the startle stimulus, as shown here) to the situation without ISI (i.e., 0 ms) might clarify this point.

### Longer Gap Durations are More Suitable for Behavioral Test Paradigms

The data discussed above mainly confirm the literature on gap duration in rats, extend our knowledge to long gap durations and provide comparable data for gerbils. Acknowledging the fact, however, that there are spectral influences on gap detection leads to a more sophisticated picture.

While the level of gap-PPI in a BBN background in rats was comparable for short (75 ms) and long (500 ms) gap durations, frequency-specific gap-PPI was, over a broad frequency range, higher with 500 ms than with 75 ms gap duration in rats. This was confirmed in the detailed analysis of underlying startle amplitudes, showing stronger inhibition of the startle amplitude with 500 ms gap duration in rats. Moreover, gap-PPI was less variable between animals with longer gap durations. Thus, our data indicate that longer gap durations are especially well suited for frequency-specific gap-PPI measurements, since frequency-specific gap duration effects are minimized. Applications of gap-PPI for tinnitus assessment in the literature used mostly short (50 ms) gaps (e.g., Turner et al., 2006; Yang et al., 2007; Nowotny et al., 2011; Berger et al., 2013). In studies on pharmacological treatments a gap duration of up to 160 ms were considered acceptable (Zou et al., 2007). Our data suggest that longer gaps are perhaps suited even better than short ones, because of a more robust gap-PPI. High and stable gap-PPI levels are necessary for behavioral tests, such as tinnitus assessment, because one tries to detect dipping levels caused by pathological hearing. When gap-PPI levels are too low (meaning near the noise level) beforehand, it is impossible to detect differences. An additional advantage of rather long gaps could be that also faint influences under investigation, such as a tinnitus percept, might become more prominent. But it could also be the other way around, that a short gap is more sensitive (can be better “filled” by a tinnitus percept). So the assumption that longer gaps not only lead to more stable gap-PPI levels, but also are more suitable for tinnitus assessment has to be further investigated. Long prepulses in the time range of several hundred milliseconds have successfully been applied in the literature in psychoacoustical test paradigms based on the ASR. Recent studies in rats used complex prepulse combinations of up to 1000 ms duration with prominent inhibitory influences on the following startle response (Fitch et al., 2008).

Gap-PPI in gerbils, on the other hand, was less dependent on spectral and temporal influences of the background noise than in rats. No differences in the amount of gap-PPI were observed between gap durations or frequencies. Long gap durations up to 1000 ms led to similar gap-PPI levels as short gaps (50 ms), however, at somewhat lower level of inhibition than rats. Since the frequency influence on gap-duration effect was minimal in gerbils, and since this species showed no relevant long-term habituation, gerbils might also make a good candidate to study tinnitus via gap-PPI, at least from these points of view.

### The Influence of Frequency on Gap-PPI is Different Between Rats and Gerbils

The general pattern regarding spectral influences in the frequency range of 4–18 kHz in gerbils and 4–20 kHz in rats taken from this study is, that in gerbils the influence of prepulses (NB and gaps) is frequency-independent, while there is a difference between NB-PPI and (frequency-dependent) gap-PPI in rats. The level of inhibition, on the other hand, can be stronger in rats (around 70–80%) than in gerbils (around 50–60%).

In more detail in rats, gap-PPI was slightly lower in the frequency range below 16 kHz and clearly reduced at 4 kHz showing strong dependency on background frequency. Interestingly, gap-PPI using pure-tone background compared to noise was much less frequency-dependent, but with a similar tendency (reduction) at the low frequency boundary. Gerbils, on the other hand, showed no frequency-dependency of gap-PPI at all.

One possible explanation for the frequency-dependency of gap-PPI in rats might be (frequency-dependent) hearing sensitivity. It is very likely that not absolute SPL itself but the sensation level is influencing the amount of gap-PPI. The different frequency-bands applied in this study probably have different sensation levels and a dependence of gap-PPI on background noise SPL has been described before Gaese et al. (2009). Especially the elevated hearing threshold at 4 kHz in rats could lead to smaller gap-PPI values, because the sensation level should be lower at this frequency (Kelly and Masterton, 1977). This might explain at least part of the lower levels of gap-PPI at frequencies below 8 kHz. This is comparable to mice (young adult, strain CBA/CaJ) showing a reduced level of gap-PPI also below 8 kHz (Ison et al., 2005), which is again consistent with the reduced hearing sensitivity in the frequency range below 8 kHz (Radziwon et al., 2009). On the other hand, a recent study determined no frequency-dependency of gap-PPI in rats, however, different NBNs and startle stimuli were used in this study (Lobarinas et al., 2015).

The lack of frequency-dependent gap-PPI in gerbils is consistent with the literature (Kiefer et al., 2015) in spite of the fact that a smaller range of frequencies was tested in gerbils compared to rats. That no frequency-dependency in the low frequency range was detected in gerbils could be also due to the fact that gerbils have higher hearing sensitivity in the low frequency region than rats (Ryan, 1976).

A rather high variability of data concerning frequency-dependency of gap-PPI was found in this study and also in the literature. Comparing data between two different rat groups tested (compare Figures 5, 9) indicates some differences in frequency-dependency, especially in the low-frequency range. Experimental effects were therefore always based on in-group comparisons. In search for the underlying mechanisms of variability we analyzed in more detail the startle amplitude underlying gap-PPI measurements in rats. Uninhibited startle amplitude (in no-gap trials) was not dependent on the frequency of background noise, and therefore was not causing the frequency-dependency of gap-PPI. Only the inhibitory effect of the gap reduced the startle amplitude in a frequency-dependent manner. This is in line with a study in mice (Lowe and Walton, 2015), finding also no frequency-dependency of the startle amplitude in no-gap trials but a frequency-dependency of gap-PPI. However, our gerbils showed a clear effect of background frequency on the uninhibited startle amplitude as it decreases with increasing frequency of background noise. This confirms prior studies indicating that startle amplitude is higher during low-frequency background noise (Schanbacher et al., 1996). Other studies in mice found also that the startle amplitude is dependent on different frequency-bands (Longenecker and Galazyuk, 2012). The assumption that the startle amplitude is not frequency-dependent is supported by the fact that neurons within the PnC that transmit startle-related activation from the cochlear nucleus (CN) to relevant motoneurons are broadly tuned and not specialized to certain frequencies assuming that this is a general characteristic of the whole startle circuit (Lingenhöhl and Friauf, 1994). Nevertheless, the differing results in literature showing no clear pattern so that it is possible that frequency-dependency of gap-PPI differs between species and is not a general phenomenon of ASR-modulation in mammals.

### Noise-Burst-PPI is Essentially Frequency-Independent in a Broad Frequency Range

We observed that NB-PPI was mainly not dependent on the prepulse frequency content over a broad frequency range in the central part of the hearing range of both, rats and gerbils. This frequency range is usually used in studies applying the ASR in behavioral test paradigms. Frequency-dependent PPI was on average as high as with BBN. PPI level in rats, however, was a bit lower only below 8 kHz and above 22 kHz. One could assume that this is simply following sensitivity as the hearing threshold of rats is actually higher at 4–6 kHz than at the most sensitive point at 8 kHz. This explanation, however, does not hold true for frequencies between 26 and 30 kHz as the sensitive hearing range of rats goes up to 40 kHz (Kelly and Masterton, 1977). Another possible explanation would focus on frequency channels: PPI is higher when the frequency of the prepulse is within the spectrum of the startle stimulus, which was in our case 1.5–20 kHz. This would only explain the decrease of inhibition at higher frequencies. It was previously shown, however, that NB-PPI is not frequency-dependent in rats (Lobarinas et al., 2015). The neuronal basis for this frequency-dependency of PPI might not be located in the main part of the PPI circuit, such as the inferior colliculus, as these areas were found to be relevant for PPI in general (Fendt et al., 2001), but not specifically for the frequency-dependency of PPI. A non-auditory structure in one of the central modulatory systems involved in regulating PPI might be frequency-dependent. Several different brain areas are involved in the regulation of PPI, e.g., by fear-potentiation, and therefore could have this function (Li et al., 2009). Different frequencies might be of different behavioral relevance, e.g., a sound from a major predator could lead to higher startle amplitudes and lower inhibition. NB-PPI, on the other hand, was shown to be frequency-dependent in mice (Carlson and Willott, 1996; Ison et al., 2005). In more detail, it was suggested that NB-PPI in mice is less dependent on the frequency of the prepulse than on the spectral contrast of the prepulse to the background (Basavaraj and Yan, 2012). Taken together, the dependency of NB-PPI on prepulse frequency seems also species specific as no consistent pattern was observed across species.

## Conclusion

Our results show that the influence of frequency on gap-PPI is different between rats and gerbils. In spite of that the following more general statements can be concluded for further applications of PPI paradigms in animal models of neurological dysfunctions and in animal psychoacoustics: (1) PPI paradigms using long gaps are more efficient as gap-PPI increases with gap duration in rats and gerbils; (2) Longer gap durations are especially important for frequency-dependent behavioral test paradigms; (3) These can be applied over a broad frequency-range, which is important e.g., for tinnitus detection tests trying to characterize in detail the phantom sound; and (4) NB-PPI is essentially frequency-independent in a broad frequency range. This allows to use variants of PPI-based behavioral tests as efficient paradigms for animal psychoacoustics.

## Author Contributions

BHG, MN, PKDP and NS: designed research, analyzed data and wrote the article; NS: performed research.

## Funding

Deutsche Forschungsgemeinschaft (GA 686/3-1); Jürgen Manchot Stiftung (personal grant to NS).

## Conflict of Interest Statement

The authors declare that the research was conducted in the absence of any commercial or financial relationships that could be construed as a potential conflict of interest. The reviewer JMP and handling Editor declared their shared affiliation, and the handling Editor states that the process nevertheless met the standards of a fair and objective review.

## Abbreviations

ASR: acoustic startle response
BBN: broad band noise
CN: cochlear nucleus
Gap-PPI: gap-prepulse inhibition
I/O: input/output
ISI: inter-stimulus interval
ITI: inter-trial interval
NBN: narrowband noise
NB-PPI: noise-burst prepulse inhibition
oct: octaves
PnC: caudal pontine reticular nucleus
PPF: prepulse facilitation
PPI: prepulse inhibition
r/f: rise/fall time
SPL: sound pressure level.
